# Non-invasive Chemokine Detection: Improved Prediction of Antibody-Mediated Rejection in Donor-Specific Antibody-Positive Renal Allograft Recipients

**DOI:** 10.3389/fmed.2020.00114

**Published:** 2020-04-09

**Authors:** Jakob Mühlbacher, Konstantin Doberer, Nicolas Kozakowski, Heinz Regele, Sümeyra Camovic, Susanne Haindl, Gregor Bond, Helmuth Haslacher, Farsad Eskandary, Jeff Reeve, Georg A. Böhmig, Markus Wahrmann

**Affiliations:** ^1^Division of General Surgery, Department of Surgery, Medical University of Vienna, Vienna, Austria; ^2^Division of Nephrology and Dialysis, Department of Medicine III, Medical University of Vienna, Vienna, Austria; ^3^Department of Pathology, Medical University of Vienna, Vienna, Austria; ^4^Department of Laboratory Medicine, Medical University of Vienna, Vienna, Austria; ^5^Alberta Transplant Applied Genomics Centre, University of Alberta, Edmonton, AB, Canada

**Keywords:** antibody-mediated rejection (ABMR), chemokines, chronic rejection, donor-specific antibodies (DSA), human leukocyte antigene (HLA), kidney, renal pathology

## Abstract

**Background:** Screening for donor-specific antibodies (DSA) has limited diagnostic value in patients with late antibody-mediated rejection (ABMR). Here, we evaluated whether biomarkers reflecting microcirculation inflammation or tissue injury—as an adjunct to DSA detection—are able to improve non-invasive ABMR monitoring.

**Methods:** Upon prospective cross-sectional antibody screening of 741 long-term kidney transplant recipients with a silent clinical course, 86 DSA-positive patients were identified and biopsied. Serum and urine levels of E-selectin/CD62E, vascular cell adhesion molecule 1 (VCAM-1), granzyme B, hepatocyte growth factor (HGF), C-C motif chemokine ligand (CCL)3, CCL4, C-X-C motif chemokine ligand (CXCL)9, CXCL10, and CXCL11 in DSA-positive recipients were investigated applying multiplexed bead-based immunoassays.

**Results:** Diagnosis of ABMR (50 patients) was associated with significantly higher levels of CXCL9 and CXCL10 in blood and urine and of HGF in blood. Overall, urinary CXCL9 had the highest diagnostic accuracy for ABMR (area under the receiver operating characteristic curve: 0.77; accuracy: 80%) and its combined evaluation with the mean fluorescence intensity of the immunodominant DSA (DSAmax MFI) revealed a net reclassification improvement of 73% compared to DSAmax MFI alone.

**Conclusions:** Our results suggest urinary CXCL9 testing, combined with DSA analysis, as a valuable non-invasive tool to uncover clinically silent ABMR late after transplantation.

## Introduction

Antibody-mediated rejection (ABMR) is a major cause of allograft failure in the long-term (1). This type of rejection is diagnosed on the basis of donor-specific antibody (DSA) detection in serum and a variety of biopsy-based morphological and molecular criteria. According to the Banff classification, detection of DSA represents an important (even though not indispensable) diagnostic criterion (2). Post-transplant detection of anti-HLA DSA is well-established to be associated with ABMR, pronounced deterioration and impaired graft survival (3). Nevertheless, it has become obvious that the presence of circulating DSA, particularly in patients with a silent clinical course, does not necessarily implicate an ongoing rejection process (4). For example, in a recent cross-sectional analysis of stable recipients recruited >6 months after transplantation, 15% of the cohort were found to be DSA-positive (5). However, out of 86 DSA-positive patients subjected to biopsy, only 44 (51%) recipients were diagnosed with ABMR (5). This result was in line with previous studies showing that in some recipients DSA in serum or other features of ABMR, such as C4d deposition on peritubular capillaries, are tied to the absence of other rejection features in biopsy (4, 6, 7). One strategy toward improved non-invasive ABMR prediction in DSA-positive subjects might be the detailed characterization of DSA properties, such as antibody binding strength or complement fixation. However, such parameters may still not precisely reflect the actual pathogenic potential of a given HLA antibody pattern (5).

Microcirculation inflammation and injury represent major characteristics of an ongoing ABMR process and may be driven by interaction of complement-fixing as well as non-complement-fixing antibodies with the microvasculature (8–10). As frequently described in the ABMR setting, cellular margination and activation, which primarily includes monocytes/macrophages and natural killer cells, are associated with an altered pro-inflammatory gene expression profile (11, 12). Hence, we proposed and probed systemically soluble E-selectin/CD62E, soluble vascular cell adhesion molecule 1 (sVCAM-1/CD106), hepatocyte growth factor (HGF) and granzyme B as indicators of membrane damage and remodeling (13–15) and the chemokines CCL3, CCL4, CXCL9, CXCL10, and CXCL11 as markers reflecting microcirculation inflammation (12, 16–21).

To test these proteins as predictors of ABMR we chose simple bead-based sandwich immunoassays that possibly can be worked up on the same flow cytometric device as the HLA beads widely used for DSA detection within 1 day. Pursuing a non-invasive approach, in this present study we analyzed prospectively sampled serum and urine specimens of 86 functionally stable long-term kidney transplant recipients with detailed clinical data and well-known DSA characteristics who underwent a protocol biopsy shortly after positive screening for DSA in the context of the interventional BORTEJECT trial (22, 23).

## Materials and Methods

### Study Design and Patients

**Figure 1:**
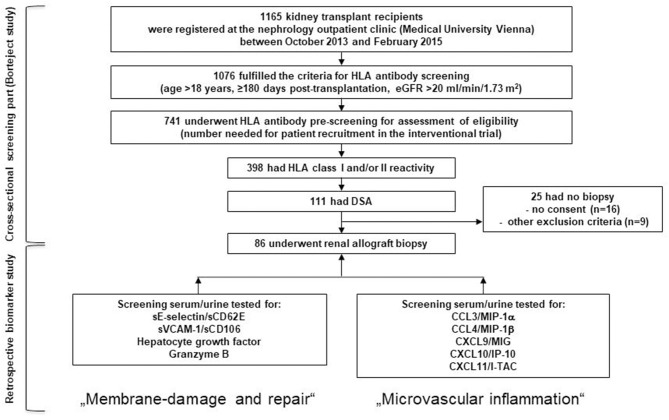
Flow chart of the screening phase of the Borteject study and the derived present biomarker study. Cross-sectional anti-HLA antibody screening of 741 renal transplant recipients identified 111 donor-specific antibody-positive patients of whom 86 underwent a protocol biopsy. The two boxes in the bottom line show the biomarkers that were retrospectively tested in those patients to predict the result of the protocol biopsies and which were grouped under the topics “membrane damage and repair” and “microvascular inflammation.” DSA, donor-specific antibody; eGFR, estimated glomerular filtration rate; HLA, human leukocyte antigen.

The objective of this study, which was performed in the context of the cross-sectional screening phase of a randomized controlled trial evaluating proteasome inhibition in late ABMR (BORTEJECT; ClinicalTrials.gov: NCT01873157), was to determine whether and to what extent a selection of biomarkers reflecting microcirculation inflammation and injury allows for the non-invasive detection of late silent ABMR. As illustrated in Figure 1, study patients were identified by systematic consecutive HLA antibody screening of 741 transplant recipients who all had a functioning renal allograft [estimated glomerular filtration rate [eGFR] (24) above 20 ml/min/1.73 m^2^] ≥180 days after transplantation (screening period from October 2013 through February 2015; nephrology outpatient clinic at the Medical University of Vienna). Patient screening revealed 111 DSA+ recipients. Of those, 86 recipients underwent protocol biopsies and were included in the study (Figure 1) (22, 23). The study was approved by the institutional ethics committee (Medical University of Vienna, EK 515/2012) and conducted in compliance with the Good Clinical Practice Guidelines, the principles of the Declaration of Helsinki 2008 and the Declaration of Istanbul.

### Biopsies

Histomorphology was evaluated on formalin-fixed paraffin-embedded sections applying standard methodology. Lesions were evaluated and scored according to the Banff 2017 classification of renal allograft pathology (2). For immunohistochemical C4d staining on paraffin sections a polyclonal anti-C4d reagent (BI-RC4D; Biomedica, Vienna, Austria) was used. For assessment of multilayering of basement membranes of peritubular capillaries (MLPTC), biopsies were evaluated by electron microscopy. Morphological results were evaluated by two experienced renal transplant pathologists (H.R. and N.K.). In addition, fractions of biopsy cores were evaluated for gene expression patterns using the Molecular Microscope Diagnostic (MMDx) platform as previously described in detail (23). Following the 2017 Banff scheme (2), ABMR was defined on the basis of histomorphological, immunohistochemical (C4d), ultrastructural (MLPTC), and serological (DSA detection) criteria as well as a thoroughly validated MMDx-based classifier for ABMR [molecular ABMR score ≥0.2; trained in a test set of 1208 biopsies (25)], respectively (5).

### Biological Material Collection and Biomarker Measurements

Serum and urine samples were collected at the time of screening. Urine was protected immediately by the addition of protease inhibitors according to the protocol of Morita et al. (26), and after centrifugation (1,890 × g, 10 min, 22°C) both supernatants, serum and urine, were aliquoted and stored at −80°C for further testing.

For quantitation of soluble VCAM-1 screening sera from 86 patients were diluted 1:200 with Universal Assay Buffer (ProcartaPlex Human Basic Kit, Thermo Fisher Scientific, Waltham, MA, USA) according to manufacturer instructions. For all other measurements (CXCL9, CXCL10, CXCL11, CCL3, CCL4, HGF, E-selectin and granzyme B) undiluted screening sera were adjusted to 10 mM EDTA to preclude false low test results due to complement interference. Samples were then measured in duplicates by a single (VCAM-1) or by multiplexed Human ProcartaPlex Simplex Immunoassays (Thermo Fisher Scientific). All urine measurements were performed in duplicates in multiplex sessions without prior EDTA adjustment or dilution of samples. All steps were carried out according to the manufacturer's protocol. Measurements and analysis of all Human ProcartaPlex Immunoassays were performed on a Luminex 200 instrument (Luminex Corp., Austin, Tx, USA). Urinary results were normalized to creatinine excretion and presented as pg (biomarker)/mg (creatinine). Urinary results were available for 83 patients. For three subjects no adequate material was available.

### HLA Antibody Detection

Patterns of HLA alloreactivity were characterized on bead-based arrays as previously described (5, 23). Briefly, for cross-sectional HLA antibody prescreening LABScreen Mixed assays (One Lambda, Canoga Park, CA, USA) were used. For further identification and characterization of DSA, patient sera were heat-inactivated (30 min, 56°C) to preclude complement interference and then tested with LABScreen Single Antigen HLA Class I and Class II flow beads (One Lambda). Threshold of positivity was set to mean fluorescent intensity (MFI) levels >1000. The immunodominant DSA (DSAmax) which is the donor-specific IgG reactivity with the highest MFI, was investigated and its value ([IgG]DSAmax) was recorded. Detection of C1q and C3d deposition on HLA beads was performed as previously described (5) and the respective MFI values of the corresponding DSAmax bead were recorded as [C1q]DSAmax and [C3d]DSAmax.

### Statistical Methods

Continuous data are given as the median and the interquartile range (IQR). Discrete data are presented as counts and percentages. Continuous data were compared by Mann-Whitney-U and dichotomous variables by Pearson's Chi square or Fisher's exact tests as appropriate. Receiver operating characteristic (ROC) analyses were performed to display the sensitivity and specificity of significant biomarkers and to determine the respective thresholds with the highest accuracy (highest sum of true-positive and true-negative predictions). Bivariate correlations were calculated using Spearman coefficient. Random forest analysis [package randomForestSRC (27)] was employed to calculate the relative importance of variables (RVI), using the permutation method. Net reclassification improvement (NRI) using the package “Hmisc” (28) was used to compare the out-of-bag predictions from various random forest models. Generally, a two-sided *P* < 0.05 was considered statistically significant. All analyses were performed using IBM SPSS Statistics Version 24 (IBM, Armonk, NY, USA) or R version 3.6.1 (https://www.r-project.org, Vienna, Austria) (29).

## Results

The study cohort consisted of 86 DSA+ recipients who were identified upon cross-sectional screening ≥180 days post-transplantation and who were all subjected to protocol biopsies (median eGFR 54 ml/min/1.73 m^2^, interquartile range [IQR]: 32–71) 5 years (median; IQR: 2.0–13.1) after transplantation. Sixty-five patients received a triple maintenance immunosuppression therapy, 21 a dual therapy. These maintenance regimens consisted of Tacrolimus (52 patients), Cyclosporine A (29 patients), mammalian target of rapamycin (mTOR, 4 patients), Belatacept (1 patient), mycophenolic acid or azathioprine (76 patients) and steroids (75 patients). Twenty-seven recipients had DSA against HLA class I, 42 against HLA class II, and 17 had DSA against both HLA class I and II antigens. While 50 of the recipients fulfilled the criteria of ABMR, 36 did not. Fifteen patients were diagnosed with active ABMR, 33 with chronic active ABMR and 2 with chronic glomerulopathy without evidence of current/recent antibody interaction with the vascular endothelium. Six patients with active and 18 patients with chronic active ABMR showed linear C4d staining in peritubular capillaries. Further patient characteristics are detailed in Table 1.

**Table 1:** Baseline demographics and patient characteristics.

	**Biopsied DSA+**	**ABMR**	**no ABMR**	
**Parameters**	***n* = 86**	***n* = 50**	***n* = 36**	***P*^a^**
**Variables recorded at the time of transplantation**				
Recipient age (years), median (IQR)	47 (36–54)	48 (34–54)	47 (39–55)	0.58
Donor age (years), median (IQR)^b^	46 (35–58)	46 (30–59)	44 (36–56)	0.76
Female recipient sex, *n* (%)	39 (45.3)	25 (50)	14 (38.9)	0.31
Live donor, *n* (%)	14 (16.3)	8 (16)	6 (16.6)	0.94
ABO-incompatible live donor transplant, *n* (%)	1 (1.2)	0 (0)	1 (2.8)	0.42
Cold ischemia time (hours), median (IQR)^c^	12 (9–17)	12 (9–18)	11 (4–15)	0.19
Prior kidney transplant, *n* (%)	25 (29.1)	15 (30)	10 (27.8)	0.82
HLA mismatch in A, B and DR, median (IQR)^d^	3 (2–4)	3 (2–3)	3 (2–4)	0.05
Latest CDC panel reactivity ≥10%, *n* (%)^e^	15 (18.5)	9 (19.1)	6 (17.6)	0.86
Preformed anti-HLA DSA, *n* (%)^f^	25 (59.5)	20 (76.9)	5 (31.3)	0.00
Induction with anti-thymocyte globulin, n (%)	28 (32.6)	22 (44)	6 (16.7)	0.01
Induction with IL-2R antibody, n (%)	28 (32.6)	11 (22)	17 (47.2)	0.01
Peri-transplant immunoadsorption, n (%)^g^	26 (30.2)	20 (40)	6 (16.7)	0.02
CDC crossmatch conversion before transplantation, n (%)	8 (9.3)	6 (12)	2 (5.6)	0.46
**Variables recorded at the time of ABMR screening**				
Recipient age (years), median (IQR)	55 (45–62)	55 (42–61)	55 (47–63)	0.58
eGFR (ml/min/1.73 m^2^), median (IQR)	54 (32–79)	44 (30–77)	58 (29–84)	0.18
Urinary protein/creatinine ratio (mg/g), median (IQR)	192 (79–445)	258 (84–1054)	167 (67–285)	0.05
No. of DSA, median (IQR)	1 (1–2)	1 (1–2)	1 (1–1)	0.09
[IgG]DSA_max_ (MFI), median (IQR)	2952 (1476–7454)	3879 (2118–10781)	1491 (1182–3462)	0.00
[C3d]DSA_max_ (MFI), median (IQR)	219 (46–2654)	414 (56–5563)	95 (36–327)	0.03
[C1q]DSA_max_ (MFI), median (IQR)	86 (30–1269)	89 (30–15820)	83 (28–257)	0.13
**Variables recorded at the time of protocol biopsy**				
Time to biopsy (years), median (IQR)	5.0 (2.0–13.1)	4.9 (2.1–13.2)	5.1 (1.6–12.7)	0.79
Time from screening to biopsy (days), median (IQR)	23 (15–41)	23 (13–36)	26 (18–45)	0.15

*ABMR, antibody-mediated rejection; DSA, donor-specific antibody; CDC, complement-dependent cytotoxicity; eGFR, estimated glomerular filtration rate; [IgG]/[C3d]/[C1q]DSA_max_, immunoglobulin G/complement split product C3d/C1q deposition of immunodominant DSA; IL-2R, interleukin 2 receptor; IQR, interquartile range; MFI, mean fluorescence intensity*.

a *Continuous data were compared by Mann–Whitney U-test, p-values of dichotomous variables were calculated by Pearson's Chi square test or Fisher's exact test as appropriate*.

b *Donor age was not recorded for 3 recipients*.

c *Cold ischemia time and ^e^CDC panel reactivity was not recorded for 5 recipients*.

d *HLA mismatch was not recorded for 1 patient*.

f *Pre-transplant DSA data were available for 42 recipients (solid-phase HLA antibody screening on the wait list was implemented at the Vienna transplant unit in July 2009)*.

g *According to our local standard, sensitized patients (until 2009: ≥40% CDC-PRA; since 2009: preformed DSA) were subjected to an earlier detailed protocol of peri-transplant immunoadsorption (30)*.

### Levels of Serum and Urinary Marker Proteins in Relation to ABMR

In a first serum analysis CXCL9, CXCL10, and HGF were the only markers showing significant differences (*p* < 0.05) between DSA+ABMR- and DSA+ABMR+ patients (Table 2, Supplementary Figure 1). After Bonferroni correction for multiple testing only CXCL9 remained significant (*p* < 0.0057, Table 2). Levels of CXCL9 were in median 276 (interquartile range [IQR]: 137–494) pg/ml vs. 412 (IQR: 277–674) pg/ml. Levels of CXCL10 were 239 (182–370) vs. 346 (221–472) pg/ml and levels of HGF 424 (307–605) vs. 525 (416–614) pg/ml, respectively.

**Table 2:** Markers in serum and urine of DSA-positive patients with and without biopsy-proven ABMR.

**Serum**			
**Parameter (in pg/ml), median (IQR)**	**ABMR- (*n* = 36)**	**ABMR+ (*n* = 50)**	***P*^**a**^**
CCL3	31 (19–47)	32 (26–70)	0.15
CCL4	46 (26–65)	49 (34–69)	0.32
CXCL9	276 (137–494)	412 (277–674)	0.002
CXCL10	239 (182–370)	346 (221–472)	0.03
CXCL11	104 (72–139)	138 (86–227)	0.06
Granzyme B	416 (301–578)	469 (329–681)	0.38
HGF	424 (307–605)	525 (416–614)	0.03
sE-selectin	44786 (36938–57619)	44608 (33122–62384)	0.92
sVCAM-1	499770 (375363–691485)	537311 (463274–651348)	0.30
**Urine**			
**Parameter (pg/mg)**^**b**^**, median (IQR)**	**ABMR- (*****n*** **=** **35)**^**c**^	**ABMR+** **(*****n*** **=** **48)**^**c**^	***P***^**a**^
CCL3	2 (1–4)	3 (1–6)	0.23
CCL4	7 (0–12)	9 (2–24)	0.15
CXCL9	14 (7–43)	47 (31–94)	<0.001
CXCL10	96 (40–177)	274 (159–375)	<0.001
CXCL11	88 (37–316)	99 (36–362)	0.44
Granzyme B	7 (0–47)	16 (1–73)	0.22
HGF	1594 (1264–1990)	1521 (1063–2031)	0.32
sE-selectin	8274 (4642–17267)	9565 (5842–19374)	0.32
sVCAM-1	398 (27–1077)	1451 (141–8040)	0.01

*ABMR, antibody-mediated rejection; CCL, chemokine (C-C motif) ligand; CXCL, chemokine (C-X-C motif) ligand; DSA, donor-specific antibody; HGF, hepatocyte growth factor; IQR, interquartile range; sE-selectin, soluble E-selectin; sVCAM-1, soluble vascular cell adhesion molecule 1*.

a *For statistical comparisons the Mann-Whitney-U test was applied. The standard p-value for significance was Bonferroni-corrected for multiple testing from 0.05 to 0.0057 with the formula 1-(1-α)^1/k^ assuming k as 9. Using this new threshold only serum CXCL9, urine CXCL9 and CXCL10 remain statistically significant*.

b *Biomarker measurement (pg/ml) was normalized to creatinine in urine (mg/ml)*.

c *Data reporting was not possible in two ABMR patients and in one non-rejecting patient*.

Subsequently we also tested our set of biomarker candidates in the patients' urine. Urinary CXCL9, CXCL10 and soluble VCAM-1 were the only biomarkers that exhibited a significant difference between ABMR- and ABMR+ patients (p levels from <0.001 to 0.01). After Bonferroni correction urinary VCAM-1 was not statistically significant anymore (requirement: *p* < 0.0057, Table 2). CXCL9 levels were in median 14 (IQR: 7–43) vs. 47 (IQR: 31–94) pg/ml, CXCL10 levels 96 (40–177) vs. 274 (159–375) and sVCAM-1 levels 398 (27–1077) vs. 1451 (141–8040) pg/mg, respectively (Table 2, Supplementary Figure 1).

Comparing serum and urinary parameters, we found a more pronounced difference in urinary CXCL9 and CXCL10 between ABMR+ and ABMR- patients than in serum CXCL9 and CXCL10 (*p*-values < 0.01–0.03 in serum analysis vs. *p*-values < 0.001 in urine analysis; Table 2). When sub-analyzing ABMR-related single lesions this finding was reflected by a more pronounced difference regarding the presence or absence of peritubular capillaritis (p ≤ 0.001). Differences were less pronounced for serum CXCL9/10 (*p* = 0.01–0.30) (Supplementary Tables 1, 2). Urinary VCAM-1 was different between patients with vs. without transplant glomerulopathy (*p* = 0.001) (Supplementary Table 2), however, this marker was omitted from further analysis because its levels correlated with proteinuria (rho = 0.617, *p* < 0.001).

### Predictive Accuracy of Serum and Urinary Biomarkers in DSA+ Recipients

In a next step we investigated the inherent ability for each parameter to associate with ABMR. For this purpose we first performed ROC analyses of all parameters (including clinical variables).

**Figure 2:**
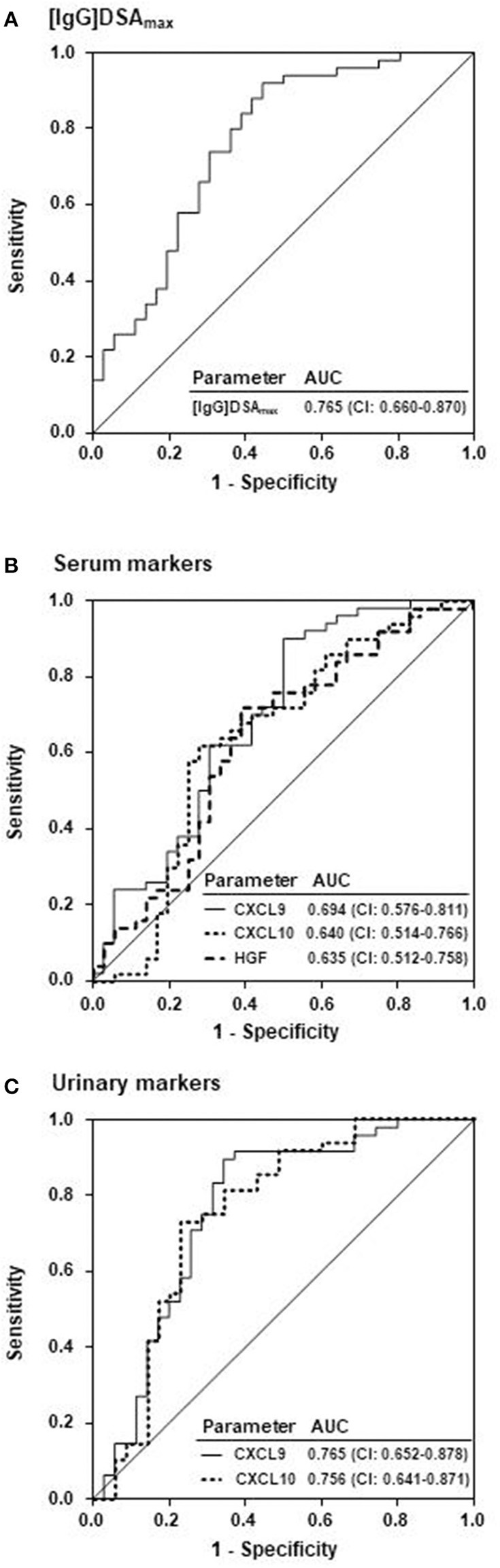
Comparison of ABMR prognosis by the MFI of the immunodominant DSA, serum and urinary biomarkers. Prediction of ABMR by receiver operating characteristic (ROC) analysis of **(A)** the MFI of the immunodominant DSA ([IgG]DSA_max_) in serum (ABMR-positive patients: 50; ABMR-negative patients: 36), of **(B)** serum CXCL9, CXCL10, and HGF and of **(C)** CXCL9 and CXCL10 normalized to creatinine in urine (ABMR-positive patients: 48; ABMR-negative patients: 35). AUC, area under the curve; CI, confidence interval.

**Figure 3:**
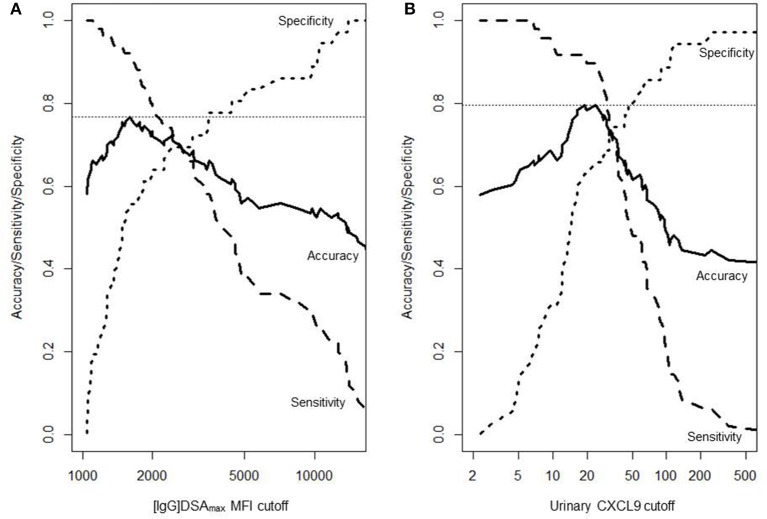
Characteristics of prediction in relation to continuous threshold values. The accuracy, sensitivity and specificity to predict ABMR in DSA-positive patients is shown for **(A)** the mean fluorescence intensity (MFI) of the immunodominant DSA (86 patients) and **(B)** urinary CXCL9 (83 patients). Unit of urinary CXCL9 is pg/mg (creatinine).

Receiver operating characteristic (ROC) curve analysis in relation to ABMR diagnosis showed the highest areas under the curve (AUCs) for urinary CXCL9 and [IgG]DSAmax (AUC: 0.77) (Figures 2A,C and Table 3). Regarding urinary CXCL9 there were two equal points of maximum accuracy (0.80), both thresholds in close proximity to each other (>18 and >22 pg/mg CXCL9). Sensitivity and specificity slightly differed in these points (18 pg/mg: 0.92 and 0.63 vs. 22 pg/mg: 0.90 and 0.66). The area under the ROC curve of urinary CXCL10 (AUC: 0.76) was almost as high as the AUC of [IgG]DSAmax and urinary CXCL9 but the maximum accuracy was only 0.75. As expected from their less pronounced distribution between ABMR and no-ABMR groups none of the serum parameters were a good predictor of ABMR and their AUC values were below 0.70 (Figure 2B and Table 3). Characteristics of predictive power (sensitivity, specificity, and accuracy) of [IgG]DSAmax and urinary CXCL9 for the full range of threshold values are presented in Figure 3.

**Table 3:** ROC analysis of clinical variables and biomarkers predicting ABMR.

**Variable**	**AUC (CI 95%)**	***P***	**Threshold with maximum accuracy**			
			**Max. accuracy**	**Sensitivity**	**Specificity**	**Threshold value**
Cold ischemia time^a^	0.59 (0.46–0.71)	0.19	0.56	0.40	0.76	15 h
eGFR	0.58 (0.46–0.71)	0.18	0.59	0.46	0.78	39 ml/min/1.73 m^2^
Protein/creatinine ratio^a^	0.62 (0.51–0.74)	0.05	0.62	0.48	0.81	300 mg/g
[IgG]DSA_max_	0.77 (0.66–0.87)	<0.001	0.77	0.92	0.56	1561 (MFI)
[C3d]DSA_max_	0.64 (0.53–0.76)	0.03	0.65	0.72	0.56	101 (MFI)
[C1q]DSA_max_	0.60 (0.48–0.72)	0.13	0.59	0.68	0.53	45 (MFI)
CXCL9 in serum	0.69 (0.58–0.81)	0.002	0.73	0.90	0.50	257 pg/ml
CXCL10 in serum	0.64 (0.51–0.77)	0.03	0.66	0.90	0.33	194 pg/ml
			0.66	0.86	0.39	203 pg/ml
HGF in serum	0.64 (0.51–0.76)	0.03	0.61	0.72	0.61	455 pg/ml
CXCL9 in urine	0.77 (0.65–0.88)	<0.001	0.80	0.92	0.63	18 pg/mg^b^
			0.80	0.90	0.66	22 pg/mg^b^
CXCL10 in urine	0.76 (0.64–0.87)	<0.001	0.75	0.92	0.51	97 pg/mg^b^
			0.75	0.81	0.66	126 pg/mg^b^
			0.75	0.73	0.77	184 pg/mg^b^

*ABMR, antibody-mediated rejection; CXCL, chemokine (C-X-C motif) ligand; DSA, donor-specific antibody; eGFR, estimated glomerular filtration rate; HGF, hepatocyte growth factor*.

a *For cold ischemia time, proteinuria and [C1q]DSA_max_ only local maxima of accuracy were considered as thresholds because absolute maxima were too extreme to produce clinically relevant cut-offs (too low specificities)*.

b *Biomarker measurement (pg/ml) was normalized to creatinine in urine (mg/ml)*.

**Figure 4:**
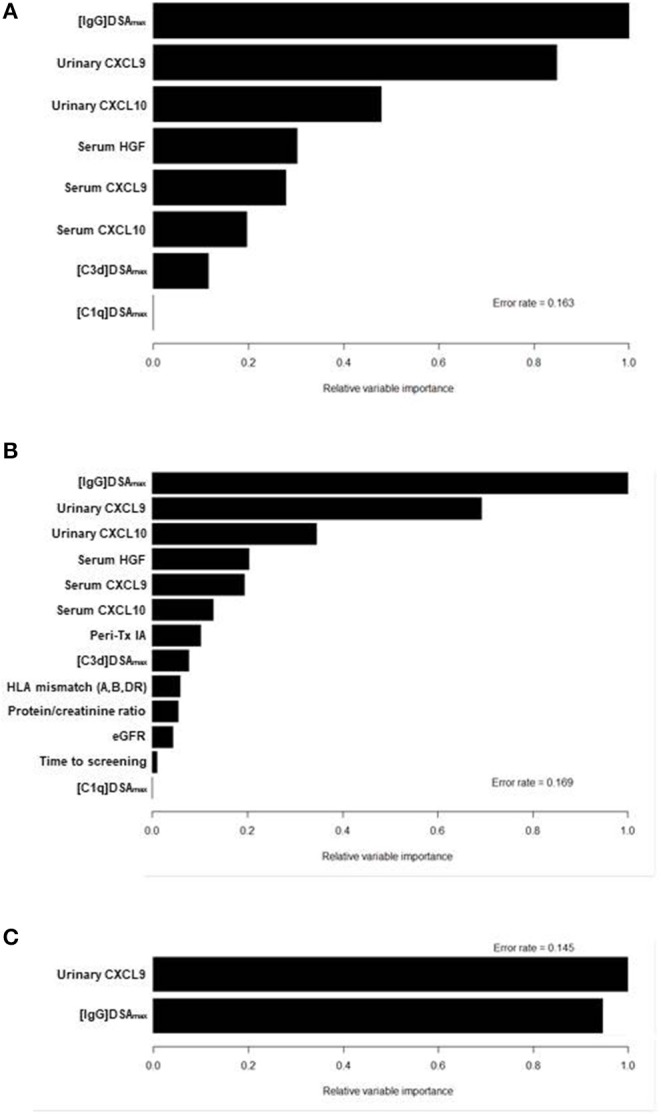
Relative variable importances (RVI) in 3 different models of random forest analysis. The RVI of a certain variable is determined by randomly shuffling the values of this particular variable in the out-of-bag-sample while keeping all other variables the same. The decrease of prediction after shuffling is a measure of the importance of this variable. In model 1 **(A)** only the following laboratory biomarkers were included: [IgG]DSA_max_ and the two derived parameters [C3d]DSA_max_ and [C1q]DSA_max_, CXCL9 and CXCL10 from serum and urine, and serum HGF. In model 2 **(B)** the laboratory biomarkers of model 1 were combined with the following clinical variables: desensitization at transplantation by immunoadsorption (IA), HLA mismatch, proteinuria, estimated glomerular filtration rate (eGFR) and time from transplantation to screening. In model 3 **(C)** variables were reduced to the two best performing variables [IgG]DSA_max_ and urinary CXCL9.

In another approach we performed a random forest analysis in order to demonstrate the relative importance of variables (RVI) contributing to ABMR prediction. In a first model we compared the relative importance of variables of laboratory parameters only (Figure 4A). The most important variable in this analysis was the MFI of the [IgG]DSAmax, followed by urinary CXCL9 and CXCL10 and the serum levels of HGF, CXCL9, and CXCL10. Levels of DSA-triggered complement fixation ([C3d]DSAmax and [C1q]DSAmax) were of the least importance here (Figure 4A). In a second model we added the clinical variables to the first model. All non-invasive laboratory markers (except for the weaker complement-fixing DSAmax characteristics) were more important than the most important clinical variable, presensitization associated with the use of peri-transplant immunoadsorption (30) (Figure 4B).

In a final random forest analysis we reduced the model to the two most important variables so far, [IgG]DSAmax and urinary CXCL9, and observed a reversed order with CXCL9 becoming the most important predictor (Figure 4C). This parallels the slightly higher accuracy of urinary CXCL9 in predicting ABMR in DSA-positive patients compared to [IgG]DSAmax (0.80 vs. 0.77; Table 3). We observed a high correlation between the variables urinary CXCL9 and urinary CXCL10 (rho = 0.9, *p* < 0.001). This suggests a supportive role of CXCL10 as an ABMR predictor during random shuffling of CXCL9 values which reduced relative importance of CXCL9 in model 1 and 2 (which both included CXCL10) compared to model 3.

### Combined Biomarkers and ABMR in DSA-Positive Patients

We then compared ABMR prediction of combined markers vs. the MFI of the immunodominant DSA alone by out-of-bag random forest predictions. The highest net reclassification improvement (NRI = 73%) was observed for the comparison of [IgG]DSAmax and urinary CXCL9 vs. [IgG]DSAmax alone which means a 73% net improvement in the samples whose classification changed with the addition of urinary CXCL9. Combining urinary CXCL10 and [IgG]DSAmax or a three-way combination of [IgG]DSAmax, CXCL9 and CXCL10 yielded lower net reclassification improvement values (46 and 60%, respectively) while all combinations of serum C-X-C motif chemokine receptor 3 (CXCR3) ligands showed NRI values below 40% and were not statistically significant (Table 4).

**Table 4:** Comparison of single and combined parameter analysis predicting ABMR in DSA-positive patients.

**Comparison**	**Net reclassification improvement (NRI) (%)**	***P***
[IgG]DSA_max_ vs. [IgG]DSA_max_ + serum CXCL9	33	0.13
[IgG]DSA_max_ vs. [IgG]DSA_max_ + serum CXCL10	27	0.21
[IgG]DSA_max_ vs. [IgG]DSA_max_ + serum CXCL9 + CXCL10	39	0.07
[IgG]DSA_max_ vs. [IgG]DSA_max_ + urine CXCL9	73	0.0003
[IgG]DSA_max_ vs. [IgG]DSA_max_ + urine CXCL10	46	0.03
[IgG]DSA_max_ vs. [IgG]DSA_max_ + urine CXCL9 + CXCL10	60	0.004

*ABMR, antibody-mediated rejection; CXCL9/10, chemokine (C-X-C motif) ligand 9/10; [IgG]DSA_max_, MFI of the immunodominant donor-specific antibody*.

## Discussion

Non-invasive diagnosis of ABMR represents an unmet need in post-transplant monitoring of renal allograft recipients. In a previous biomarker analysis we could demonstrate that the MFI value of the immunodominant donor-specific anti-HLA antibody ([IgG]DSAmax) was a superior predictor of ABMR in long-term transplanted DSA-positive recipients compared to DSAmax derived complement-fixing parameters like [C1q]DSAmax or [C3d]DSAmax (5). The aim of the present study, which was conducted in the same cohort, was to find a marker that is able to reflect microcirculation inflammation or signs of membrane damage since both are essential elements of ABMR diagnosis according to Banff consensus (2). In our present study a single-parameter prediction by the MFI of [IgG]DSAmax exhibited an accuracy of 77% while the marker urinary CXCL9 showed 80%. Introducing a combination of both tests revealed a 73% net improvement in accuracy compared to prediction by MFI of [IgG]DSAmax alone. These results of the present study indicate that measurement of urinary CXCL9, especially in conjunction with DSA analysis, is able to improve the non-invasive diagnosis of ABMR. Such an improved diagnostic test could allow for identification of DSA-positive patients with a clinically silent ABMR in outpatient routine management who should be further subjected to a more invasive biopsy for a more detailed diagnosis.

CXCL9, CXCL10, and CXCL11 are chemokines which are able to induce chemotaxis in CD4+ Type-1 helper (Th1) and CD8+ cytotoxic lymphocytes as well as in natural killer cells and natural killer T cells, directing them to sites of infection and inflammation. All three chemokines are induced by interferon-γ (IFN-γ) and share CXCR3 as a common receptor. Although their function may thus appear to be redundant, they are differentially regulated by other stimuli of synthesis than IFN-γ, post-translational processing and finally degradation by various matrix metalloproteases (31, 32). CXCL9 and CXCL10 have previously attracted attention when their urinary levels, normalized to creatinine, were shown to be increased in ABMR as well as T cell-mediated rejection (TCMR) (33–35). In particular, the study of Rabant et al. (35) is of note which led to similar conclusions as our present study, namely that measurement of urinary CXCL10 adds diagnostic value to the measurement of the immunodominant DSA MFI, albeit their study design started from other baseline conditions. The authors of this study tried to predict rejections in indication biopsies and found a large number of non-rejecting patients as well as cases of ABMR, TCMR and mixed rejections while our protocol biopsy study clearly focused on DSA-positive patients with virtually no cases of TCMR (one patient with pure TCMR and 5 patients with borderline rejection and ABMR and 4 patients with pure borderline rejection). This could be the reason for a slight difference between the two studies: while the study of Rabant et al. (35) highlighted CXCL10, all of our analyses—no matter whether derived from serum or urine—pinpoint CXCL9 to be the preferable protein biomarker for the prediction of late ABMR among DSA-positive patients.

One strength of the BORTEJECT study may be its well-defined patient population which is at the same time a minor disadvantage in our derivative biomarker survey: patients with other possible causalities of inflammation were not included (like an active malignant disease or infections with virus, fungi and/or bacteria) or were infrequent in our cohort (TCMR). Therefore, our findings cannot answer the question whether CXCL9 is able to discriminate “true positive” patients with antibody-mediated rejection from possibly “false positive” patients with inflammation from any other cause. Consequently such eventualities should be ruled out by conventional clinical practices because they are likely to interfere with diagnosis of chronic ABMR by detection of CXCL9 (36, 37).

It appears that the markedly improved prediction by urinary CXCR3 ligands—compared to the serum analogs—is mainly borne by an improved prediction of peritubular capillaritis rather than of glomerulitis. A possible pathophysiological explanation for this could be that in an IFN-γ -driven renal microcirculation inflammation setting not only endothelial but also tubular epithelial cells are a major source of CXCR3 ligand synthesis which may secrete significant amounts in both basal and apical directions (21). The amount adjusted to the former direction may then contribute to the observed peritubular capillaritis by chemotaxis and the amount directed to the latter direction could be reasoned by the only recently discovered direct microbicidal activity of CXCL9 and CXCL10 (38–40). These apically secreted amounts of chemokines, which are likely to act prophylactically toward potential microbial invaders in this inflammatory situation, represent an excellent opportunity for exploitation as a diagnostic test by collecting and testing the urine.

Discussing possible pathophysiologic mechanisms of chronic ABMR, our finding of increased expression of CXCR3 ligands is in line with other recent findings which suggest that NK cells (which together with T cells are the main expression site of CXCR3) may play a major role in antibody-mediated rejection (41–43). However, although such an interpretation of our data seems likely, it should be tried only with caution because the design of this study was primarily shaped to find biomarkers not to reveal pathophysiologic mechanisms.

In summary we suggest CXCL9 (but not CXCL10) testing in urine as an adjunct test to immunodominant DSA characterization in serum for the prediction of late antibody-mediated rejection in clinically well-presenting, but DSA-positive long-term kidney transplant patients.

## Data Availability Statement

The dataset for this article is not publicly available because: this is not covered by the votum of the ethics committee and might potentially infringe the privacy rights of patients. Requests to access the datasets should be directed to Markus Wahrmann, markus.wahrmann@meduniwien.ac.at.

## Ethics Statement

The studies involving human participants were reviewed and approved by Ethikkommission der Medizinischen Universität Wien Borschkegasse 8b/61090 Wien, Austria. The patients/participants provided their written informed consent to participate in this study.

## Author Contributions

JM, JR, GAB, and MW participated in research design, performance of research, data analysis, and writing of the paper. KD, NK, HR, SC, and SH performance of research and data analysis. GB and HH participated in data analysis. FE participated in research design and data analysis. All authors read and approved the final manuscript.

### Conflict of Interest

The authors declare that the research was conducted in the absence of any commercial or financial relationships that could be construed as a potential conflict of interest.
