# Supplementary material for: Non-invasive Chemokine Detection: Improved Prediction of Antibody-Mediated Rejection in Donor-Specific Antibody-Positive Renal Allograft Recipients
Source: Front Med (Lausanne). 2020 Apr 9;7:114. doi: 10.3389/fmed.2020.00114 (PMC7160229; doi:10.3389/fmed.2020.00114)
Supplement: Supplementary file 1 [file Data_Sheet_1.PDF]

Supplementary Material

1 Table of Contents

Suppl. Table 1 ………………………………………………………………………… Page 2

Suppl. Table 2 ………………………………………………………………………… Page 3

Suppl. Figure 1 ……………………………………………………………………... Page 4

Fig. Legend Suppl. Figure 1 ……………………………………………………...….. Page 5

2 Supplementary Figures and Tables

2.1 Supplementary Tables

Supplementary Table 1. Serum analysis of 86 DSA-positive patients with and without biopsy-proven glomerulitis, peritubular capillaritis, transplant glomerulopathy, multilayering of basement membranes and MMDx score

Parameter (in pg/ml), median (IQR)	CXCL9	CXCL10	HGF
**Glomerulitis (g)**			
g- (n=41)[a]	265 (140-483)	236 (179-382)	442 (316-570)
g+ (n=41)[a]	401 (319-620)	352 (236-482)	551 (458-658)
p value	<0.01	<0.01	<0.01
**Peritubular capillaritis (ptc)**			
ptc- (n=46)[b]	307 (164-550)	265 (199-409)	434 (332-606)
ptc+ (n=38)[b]	444 (326-603)	335 (216-472)	549 (459-658)
p value	0.01	0.30	0.02
**Transplant glomerulopathy (cg)**			
cg- (n=52)[c]	314 (215-494)	268 (194-421)	470 (344-619)
cg+ (n=31)[c]	454 (307-731)	341 (213-399)	529 (389-605)
p value	0.01	0.53	0.36
**Immunohistochemical detection of C4d**			
C4d- (n=60)	334 (213-526)	268 (201-391)	470 (350-606)
C4d+ (n=26)	464 (276-674)	361 (216-562)	528 (361-658)
p value	0.03	0.06	0.39
**Molecular microscope ABMR score (≥ 0.2)**			
MM_ABMR- (n=38)[d]	261 (138-490)	230 (179-380)	420 (297-567)
MM_ABMR+ (n=45)[d]	401 (295-622)	341 (232-482)	529 (458-648)
p value	<0.01	<0.01	<0.01
**Multilayering of basement membranes of peritubular capillaries (MLPTC)**			
MLPTC- (n=39)[e]	294 (154-488)	248 (180-323)	445 (340-568)
MLPTC+ (n=44)[e]	401 (277-643)	371 (223-553)	549 (434-631)
p value	<0.01	0.001	0.04

ABMR, antibody-mediated rejection; CXCL, chemokine (C-X-C motif) ligand; DSA, donor-specific antibody; HGF, hepatocyte growth factor; IQR, interquartile range.

[a] Glomerulitis was not graded in 4 patients. [b] Ptc was not scored in 2 patients. [c] Transplant glomerulopathy grading was missing in 3 patients. [d] MMDx was not performed in 3 cases. [e] MLPTC was not analyzed in 3 patients.

Supplementary Table 2. Urine analysis of 83 DSA-positive patients with and without biopsy-proven glomerulitis, peritubular capillaritis, transplant glomerulopathy, multilayering of basement membranes and MMDx score

**Parameter (in pg/mg), median (IQR)[a]**	**CXCL9**	**CXCL10**	**sVCAM-1**
**Glomerulitis (g)**			
g- (n=39)[b]	16 (10-46)	120 (61-303)	448 (33-1511)
g+ (n=40)[b]	46 (30-94)	256 (119-375)	1451 (102-8910)
p value	<0.01	0.01	0.04
**Peritubular capillaritis (ptc)**			
ptc- (n=44)[c]	18 (10-48)	119 (61-295)	370 (28-2099)
ptc+ (n=37)[c]	60 (33-96)	275 (179-425)	1636 (194-8710)
p value	<0.001	0.001	0.02
**Transplant glomerulopathy (cg)**			
cg- (n=51)[d]	28 (12-65)	166 (67-303)	329 (27-1511)
cg+ (n=29)[d]	44 (30-88)	266 (115-370)	2295 (395-9438)
p value	0.05	0.07	0.001
**Immunohistochemical detection of C4d**			
C4d- (n=58)	31 (12-80)	167 (79-340)	565 (43-2390)
C4d+ (n=25)	44 (30-78)	245 (133-471)	1266 (269-7930)
p value	0.11	0.04	0.16
**Molecular microscope (MM) ABMR score (≥ 0.2)**			
MM_ABMR- (n=36)[e]	15 (8-49)	100 (40-266)	497 (36-1515)
MM_ABMR+ (n=44)[e]	45 (31-96)	270 (159-400)	715 (102-6419)
p value	<0.001	<0.001	0.15
**Multilayering of basement membranes of peritubular capillaries (MLPTC)**			
MLPTC- (n=38)[f]	22 (9-68)	122 (48-287)	335 (14-1512)
MLPTC+ (n=42)[f]	43 (27-80)	234 (117-374)	1132 (151-6870)
p value	0.02	0.01	0.02

ABMR, antibody-mediated rejection; CXCL, chemokine (C-X-C motif) ligand; DSA, donor-specific antibody; IQR, interquartile range; sVCAM-1, soluble vascular cell adhesion molecule 1.
[a] Biomarker measurement (pg/ml) was normalized to creatinine in urine (mg/ml). [b] Glomerulitis was not graded in 4 patients. [c] Ptc was not scored in 2 patients. [d] Transplant glomerulopathy grading was missing in 3 patients. [e] MMDx was not performed in 3 cases. [f] MLPTC was not analyzed in 3 patients.

2.2 Supplementary Figure 1

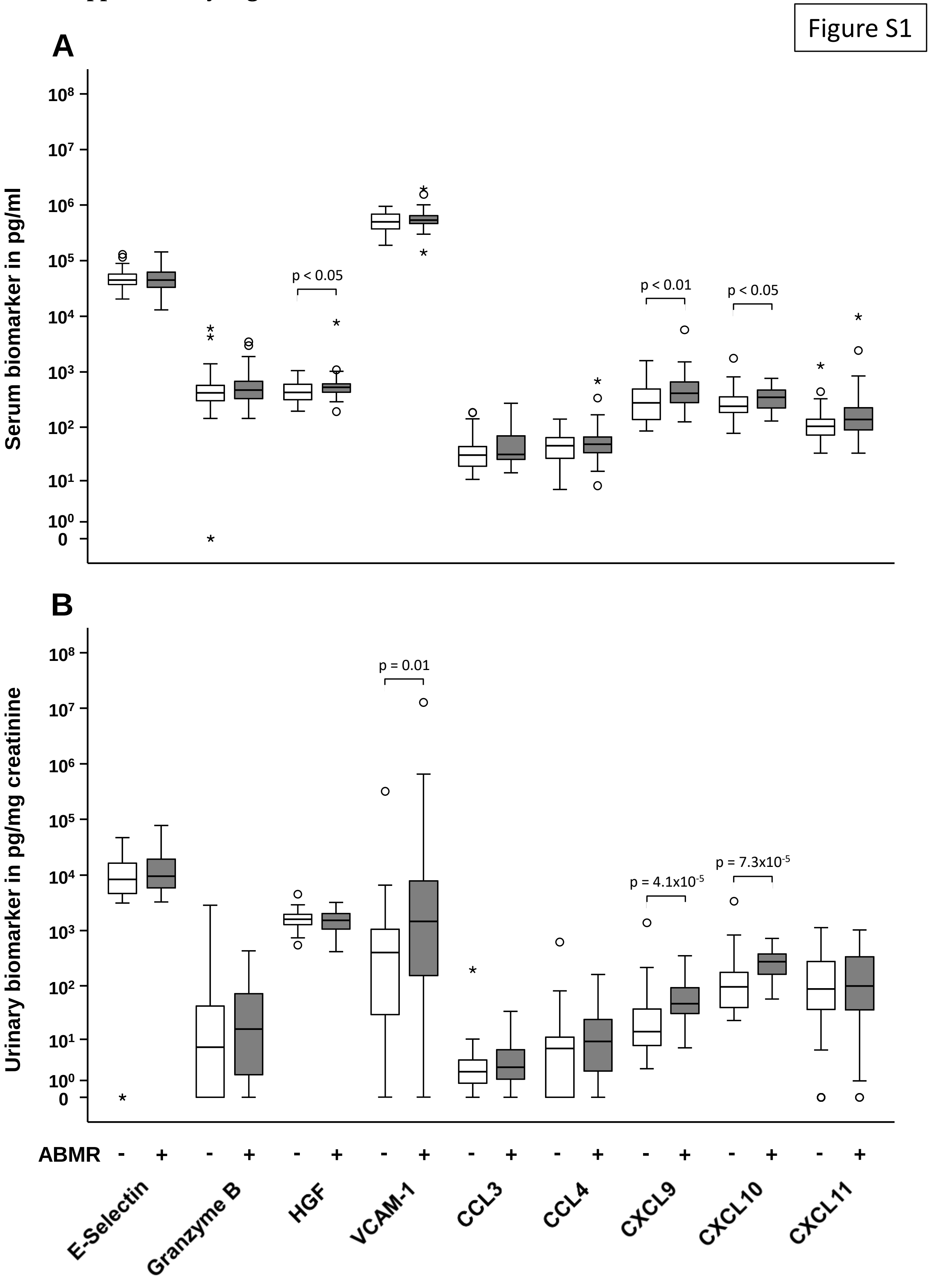

Supplementary Figure 1. Distribution of serum and urinary biomarkers between ABMR negative and ABMR-positive long-term transplanted patients with positive DSA finding. (A) Serum analysis of 50 ABMR-positive (gray boxes) and 36 ABMR-negative patients (open boxes). (B) Urine analysis of 48 ABMR-positive (gray boxes) and 35 ABMR-negative patients (open boxes). Urinary biomarker results were normalized to creatinine in the same specimen. Box plots indicate the median and IQR (outliers: circles, extreme outliers: asterisks). For statistical comparisons the Mann-Whitney-U test was applied.
